# Utilizing multimodal radiomics technology from cervical MRI for diagnosis of cervical spinal cord injury and spinal cord concussion

**DOI:** 10.1038/s41598-024-69784-8

**Published:** 2024-08-12

**Authors:** Zhigang Pan, Weipeng Hu, Zhangsheng Dai, Yuanzhe Li, Zhongning Fang, Canfang Shen, Zekai Chen, Kaibin Fang

**Affiliations:** 1 Department of Orthopaedic Surgery, The Second Affiliated Hospital of Fujian Medical University, No.34, Zhongshanbeilu, Quanzhou, 36200 Fujian China; 2 Department of Neurosurgery, The Second Affiliated Hospital of Fujian Medical University, Quanzhou, 362000 China; 3 Department of CT/MRI, The Second Affiliated Hospital of Fujian Medical University, Quanzhou, 362000 China; 4 Fujian Medical University, Fuzhou, 361000 China; 5 Jinjiang General Hospital, Quanzhou, 362000 China; 6 Shishi General Hospital, Quanzhou, 362000 China

**Keywords:** Cervical magnetic resonance imaging, Radiomics, Multimodal, Spinal cord injury, Spinal cord concussion, Neurological disorders, Biomarkers, Medical research

## Abstract

The primary aim of this study is to assess the viability of employing multimodal radiomics techniques for distinguishing between cervical spinal cord injury and spinal cord concussion in cervical magnetic resonance imaging. This is a multicenter study involving 288 patients from a major medical center as the training group, and 75 patients from two other medical centers as the testing group. Data regarding the presence of spinal cord injury symptoms and their recovery status within 72 h were documented. These patients underwent sagittal T1-weighted and T2-weighted imaging using cervical magnetic resonance imaging. Radiomics techniques are used to help diagnose whether these patients have cervical spinal cord injury or spinal cord concussion. 1197 radiomics features were extracted for each modality of each patient. The accuracy of T1 modal in testing group is 0.773, AUC is 0.799. The accuracy of T2 modal in testing group is 0.707, AUC is 0.813. The accuracy of T1 + T2 modal in testing group is 0.800, AUC is 0.840. Our research indicates that multimodal radiomics techniques utilizing cervical magnetic resonance imaging can effectively diagnose the presence of cervical spinal cord injury or spinal cord concussion.

## Introduction

Acute spinal cord injury (SCI) can cause significant sensory, motor, and autonomic impairments, potentially leading to serious outcomes like paralysis and respiratory failure^1^. Spinal cord concussion is a form of spinal cord injury characterized by a reversible functional disturbance that manifests following spinal cord trauma^2^. One defining feature of this injury is the swift and full restoration of spinal cord function below the level of the injury^3^. Unlike other types of spinal cord injuries, patients with spinal cord concussion often do not require surgical treatment, and some studies even suggest that hormone therapy is not necessary for these patients^4^. However, for patients with other types of spinal cord injuries, early intervention is crucial for their prognosis^5^. Cervical magnetic resonance imaging (MRI) is considered to be useful for early diagnosis of spinal cord injury and for predicting neurological recovery after cervical spinal cord injury by evaluating intraspinal hemorrhage and lesion length^6^.

However, not all patients without positive imaging findings can be diagnosed with spinal cord concussion. Some patients may initially have negative MRI results following injury, and it is only after 72 h of follow-up MRI that imaging abnormalities can be detected^7^. This phenomenon is commonly observed in spinal cord injury without radiographic abnormality(SCIWORA), who may require follow-up MRI in a few days^8^. Distinguishing between spinal cord concussion and true SCIWORA on MRI in the early stages can be challenging, but the transient nature of symptoms in spinal cord concussion contrasts with the persistent symptoms seen in SCIWORA, aiding in diagnosis^9^. Early intervention with conservative treatments like steroids or surgical procedures is believed to offer significant benefits to spinal cord injury patients, particularly those with SCIWORA^10,11^. This approach differs from the management of spinal cord concussion. Therefore, timely diagnosis of patients presenting with spinal cord injury symptoms, and differentiation between spinal cord injury and spinal cord concussion, holds crucial clinical importance in guiding treatment decisions.

To facilitate early diagnosis of spinal cord injury and spinal cord concussion through cervical magnetic resonance imaging, artificial intelligence technologies such as deep learning and machine learning have been employed^12,13^. Nevertheless, there have been no studies utilizing radiomics techniques to ascertain the presence of spinal cord injury in patients. Radiomics involves extracting actionable data from medical images and analyzing it to aid in diagnosis, predict prognosis, and provide clinical decision support, all with the aim of advancing precision medicine^14^.

In this study, the authors endeavored to utilize multimodal radiomics techniques based on cervical magnetic resonance imaging for the diagnosis of spinal cord injury and spinal cord concussion.

## Materials and methods

### Participants in the study and development of clinical models.

**Figure 1:**
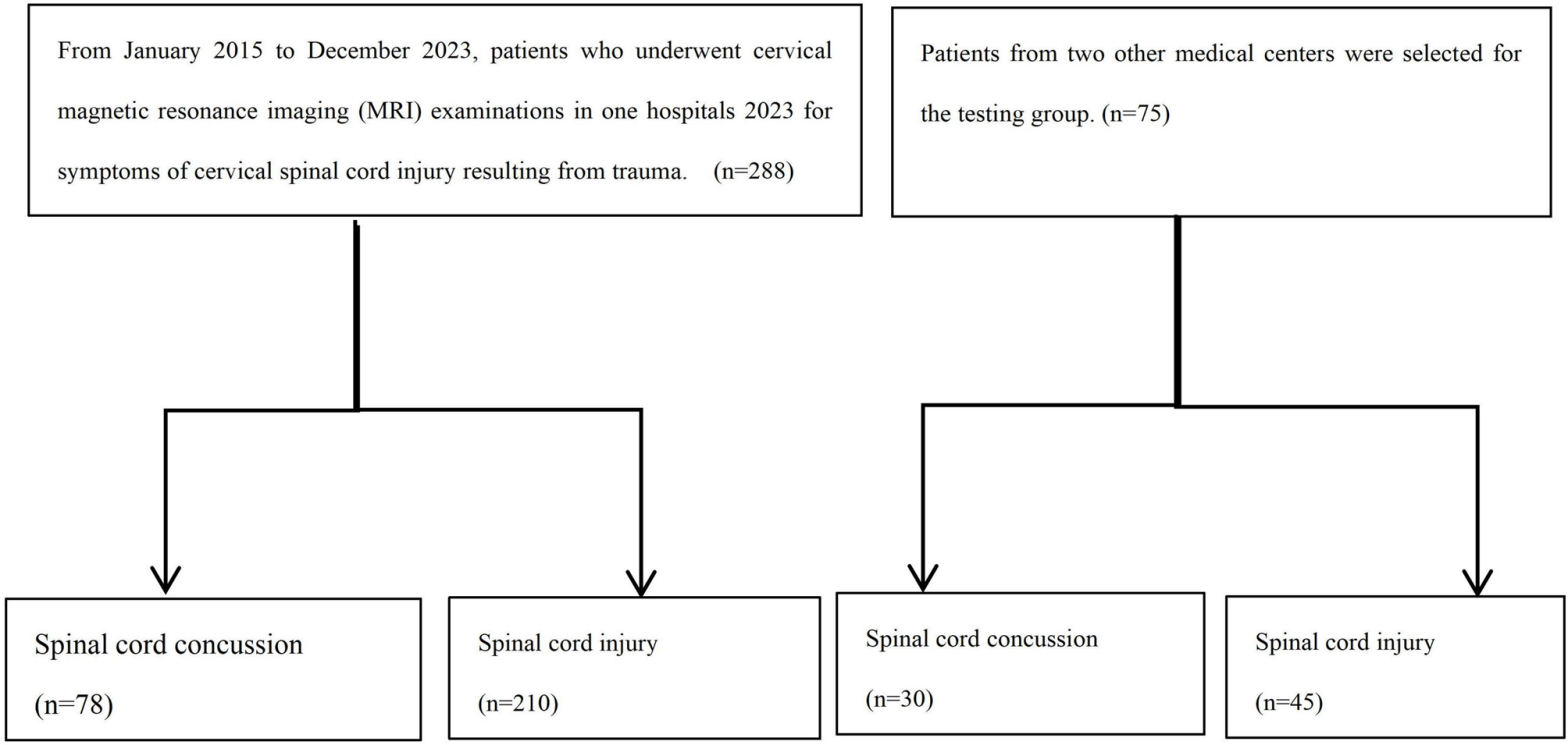
The process of patient inclusion.

This study retrospectively analyzed adult patients who underwent cervical magnetic resonance imaging (MRI) examinations in three large hospitals from January 2015 to December 2023 for symptoms of cervical spinal cord injury resulting from trauma. The MRIs were conducted within 72 h of the injury, and the study also retrieved information on symptom recovery within the same timeframe from the medical records. Patients with a history of neck surgery, artifacts affecting radiomics feature extraction, and those who underwent emergency surgery within 72 h were excluded from the study. Approval for this study was obtained from the Hospital Institutional Review Committee, and it was conducted in accordance with the principles of the Helsinki Declaration. A total of 288 patients from a large medical center were chosen for the training group, while 75 patients from two other medical centers were selected for the testing group. During the training phase, there were 78 patients diagnosed with spinal cord concussion and 210 patients with spinal cord injury. In the test group, there were 30 patients with spinal cord concussion and 45 patients with spinal cord injury. The patient inclusion process is shown in Fig. 1.

### Image preprocessing

**Figure 2:**
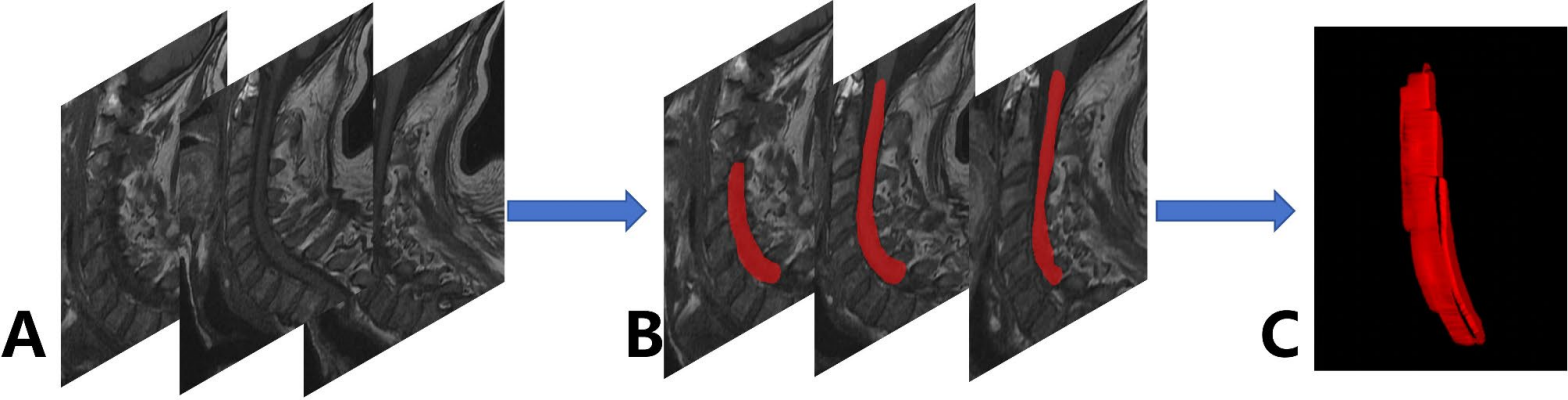
The process of outlining ROI. (**A**) Initial image. (**B**) Sketching. (**C**) Production of 3D models.

The regions of interest were specifically chosen to include sagittal T1-weighted images and sagittal T2-weighted images obtained from cervical magnetic resonance imaging. The reconstruction of images and the precise delineation of the region of interest (ROI) were executed utilizing the ITK SNAP software^15^. The patient’s cervical spinal cord has been comprehensively delineated, encompassing the entire length of the spinal cord from the first cervical vertebra to the posterior seventh cervical vertebra. The process of outlining ROI is shown in Fig. 2.

A neurosurgeon possesses expertise in outlining regions of interest (ROIs). To assess the intra- and inter-observer variability, the intraclass correlation coefficient (ICC) was employed. Initially, one researcher delineated the ROI, and subsequently, another researcher, with over 10 years of neurosurgical experience, randomly selected thirty cases to independently re-delineate the ROI. Both researchers were blinded to each other’s delineation outcomes. Utilizing the thirty cases with delineated ROIs, the ICC was calculated to evaluate the degree of agreement. Only radiomic features exhibiting ICC values exceeding 0.9 for both datasets were deemed reliable and chosen for further analysis.

### Radiomics feature extraction

The feature extraction process was carried out using the Pyradiomics Module (https://github.com/Radiomics/pyradiomics). To enrich the array of derived images, diverse filters, including Laplacian of Gaussian filters and wavelets, were utilized. All radiomic features were categorized into seven groups: shape-based features, first-order features, gray-level dependence matrix (GLDM) features, gray-level size zone matrix (GLSZM) features, neighboring gray-tone difference matrix (NGTDM) features, gray-level run-length matrix (GLRLM) features, and gray-level co-occurrence matrix (GLCM) features.

### Feature selection

To identify the most relevant features associated with cervical Spinal Cord Injury, a rigorous feature selection process was implemented. Initially, the U test (p < 0.05) was used to identify features that exhibited significant differences between the Spinal Cord Injury and Spinal Cord Concussion groups. Additionally, features with intra-class correlation (ICC) coefficients lower than 0.9 were excluded from this step. This approach effectively reduced the number of features while maintaining their predictive power. To address multicollinearity, Pearman correlation analysis was conducted to examine the relationships between features. Calculation of correlation coefficients allowed for identification of feature pairs with values ≥ 0.9 or ≤ -0.9. In such cases, only the feature demonstrating superior diagnostic performance was retained, preventing redundancy within the model introduced by highly correlated features. The method of minimum redundancy and maximum correlation (mRMR) was applied to identify the 20 most significant features from the given set of features^16^. To further refine the feature set, the least absolute shrinkage and selection operator (LASSO) logistic regression technique was employed^17^. This technique allows for variable selection while penalizing coefficients, ultimately producing a more refined and robust feature set.

### Machine learning models

For the analysis, the multilayer perceptron was chosen as the radiomics feature selector. The multilayer perceptron (MLP), a quintessential neural network model, is constructed from an array of neuron layers. Its architectural and functional attributes position it as a pivotal element within the realm of deep learning^18^. The MLP demonstrates commendable performance across a diverse range of tasks, including image and text classification, as well as prediction and regression problems^19^. The MLP model used in the manuscript consists of three layers: an input layer, a hidden layer, and an output layer. The input layer has 10 nodes, corresponding to the 10 features used in the model. The hidden layer has 64 nodes, and the output layer has 1 node, representing the binary classification output (0 or 1). The loss function used in the model is the Mean Squared Error (MSE) loss function. MSE is a common choice for regression problems, and in this case, it is adapted for binary classification by minimizing the difference between the predicted probability and the actual label (0 or 1). The radiomics features selected in the experiment would be chosen as the input data for the input layer. The output of the model is a binary classification label, where 0 represents Spinal Cord Concussion and 1 represents Cervical Spinal Cord Injury. The learning rate used in the model is 0.001. The optimization technique used in the model is Adam, an adaptive learning rate method. Adam combines the idea of momentum (gradient descent with momentum) and RMSProp, which is a method for efficiently adapting the learning rate of each parameter.

### Statistical analysis

For the analysis, leverage Python-based statistical libraries such as Statsmodels, NumPy, Pandas, and SciPy. Assess the predictive model’s efficacy by calculating the Area Under the Curve (AUC) and determine the 95% confidence interval (CI) of the AUC using the bootstrap method with 1000 resampling intervals^20^. To comparatively evaluate the AUC across various models, employ the DeLong test to statistically gauge the divergence in performance metrics between them^21^.

### Ethical approval

This retrospective study was approved by ethics committee of The Second Affiliated Hospital of Fujian Medical University(study no. IRB_2023.279). All methods were carried out in accordance with relevant guidelines and regulations.Written informed consent was waived by the ethics committee of The Second Affiliated Hospital of Fujian Medical University.As this is a retrospective study, the patient’s imaging data were desensitized before the study. Only the extracted feature area was retained as the original data, and no patient information was exposed. Therefore, after application, the informed consent form was not obtained.

### Informed consent

After our application, the informed consent was waived by the Ethics Committee of our hospital.

## Results

### Radiomics feature extraction

1197 radiomics features were extracted for each modality of each patient. Among them, there are 234 features of the firstorder type, 286 features of the glcm type, 182 features of the gldm type, 208 features of the glrlm type, 208 features of the glszm type, 65 features of the ngtdm type, and 14 features of the shape type.

### Assessing the utility of radiomics analysis derived from T1-weighted cervical MRI for discriminating between SCC and SCI

**Figure 3:**
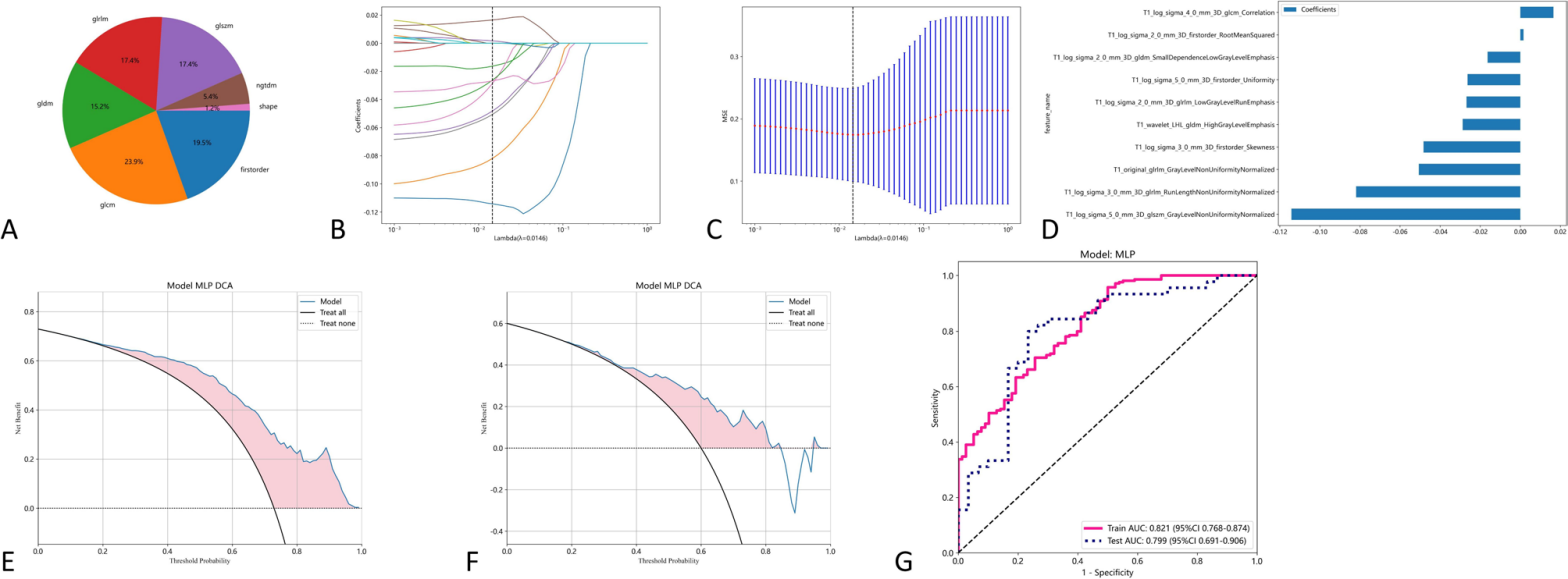
The effectiveness of the T1 modal. (**A**) Extracted radiomics features. (**B**, **C**) using radiometric methods such as LASSO regression for feature screening. (**D**) Selected radiomics features and feature weights. (**E**) Use MLP machine learning models and filtered features to distinguish the DCA curves of SCC and SCI in the training group. (**F**) Use MLP machine learning models and filtered features to differentiate the DCA curves of SCC and SCI in the test group. (**G**) Use MLP machine learning models and filtered features to distinguish the AUC curves of SCC and SCI.

After screening, 10 radiomics features based on sagittal T1 weighted images of cervical magnetic resonance imaging were extracted. The accuracy of using these features to distinguish spinal cord injury and spinal cord concussion in train group is 0.830, AUC is 0.821. The accuracy of using these features to distinguish spinal cord injury and spinal cord concussion in testing group is 0.773, AUC is 0.799. The radiomics feature weights selected based on the T1 modality of cervical magnetic resonance imaging are presented in Table 1. The effectiveness of using radiomics features based on T1 weighted phase to distinguish SCI and SCC is shown in Fig. 3 and Table 2.

**Table 1:** Radiomics features selected for the T1 radiomics model.

Image type	Feature class	Feature name	LASSO coefficient (β)
log_sigma_5_0_mm_3D	glszm	GrayLevelNonUniformityNormalized	− 0.114217
log_sigma_2_0_mm_3D	gldm	SmallDependenceLowGrayLevelEmphasis	− 0.016304
log_sigma_2_0_mm_3D	firstorder	RootMeanSquared	0.001664
wavelet_LHL	gldm	HighGrayLevelEmphasis	− 0.028719
origina	glrlm	GrayLevelNonUniformityNormalized	− 0.050644
log_sigma_3_0_mm	glrlm	RunLengthNonUniformityNormalized	− 0.081924
log_sigma_5_0_mm	firstorder	Uniformity	− 0.026292
log_sigma_3_0_mm	firstorder	Skewness	− 0.048261
log_sigma_4_0_mm	glcm	Correlation	0.016513
log_sigma_2_0_mm	glrlm	LowGrayLevelRunEmphasis	− 0.026846

**Table 2:** The efficacy of multimodal radiomics techniques in distinguishing spinal cord injury and spinal cord concussion.

model_name	Accuracy	AUC	95% CI	Sensitivity	Specificity	PPV	NPV	Precision	Recall	F1	Threshold	Task
T1	0.830	0.821	0.7679–0.8743	0.952	0.500	0.837	0.796	0.837	0.952	0.891	0.544	Train
T1	0.773	0.799	0.6907–0.9063	0.778	0.767	0.833	0.697	0.833	0.778	0.805	0.796	Test
T2	0.750	0.844	0.7928–0.8948	0.714	0.846	0.926	0.524	0.926	0.714	0.806	0.768	train
T2	0.707	0.813	0.7144–0.9108	0.622	0.833	0.848	0.595	0.848	0.622	0.718	0.852	Test
T1 + T2	0.830	0.902	0.8629–0.9415	0.838	0.808	0.921	0.649	0.921	0.838	0.878	0.766	Train
T1 + T2	0.800	0.840	0.7415–0.9385	0.844	0.733	0.826	0.759	0.826	0.844	0.835	0.773	Test

### Assessing the utility of radiomics analysis derived from T2-weighted cervical MRI for discriminating between SCC and SCI

**Figure 4:**
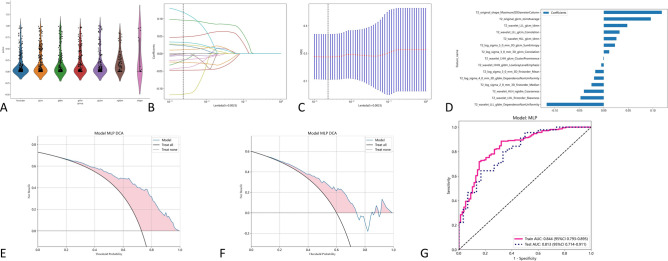
The effectiveness of the T2 modal. (**A**) Extracted radiomics features. (**B**, **C**) using radiometric methods such as LASSO regression for feature screening. (**D**) Selected radiomics features and feature weights. (**E**) Use MLP machine learning models and filtered features to distinguish the DCA curves of SCC and SCI in the training group. (**F**) Use MLP machine learning models and filtered features to differentiate the DCA curves of SCC and SCI in the test group. (**G**) Use MLP machine learning models and filtered features to distinguish the AUC curves of SCC and SCI.

After screening, 15 radiomics features based on sagittal T2 weighted images of cervical magnetic resonance imaging were extracted. The accuracy of using these features to distinguish spinal cord injury and spinal cord concussion in train group is 0.750, AUC is 0.844. The accuracy of using these features to distinguish spinal cord injury and spinal cord concussion in testing group is 0.707, AUC is 0.813. The radiomics feature weights selected based on the T1 modality of cervical magnetic resonance imaging are presented in Table 3. The effectiveness of using radiomics features based on T2 weighted phase to distinguish SCI and SCC is shown in Fig. 4 and Table 2.

**Table 3:** Radiomics features selected for the T2 radiomics model.

Image type	Feature class	Feature name	LASSO coefficient (β)
log_sigma_5_0_mm_3D	glcm	SumEntropy	+ 0.022896
log_sigma_2_0_mm_3D	firstorder	Median	− 0.024891
log_sigma_4_0_mm_3D	gldm	DependenceNonUniformity	− 0.019686
wavelet_LLL	glcm	Idmn	+ 0.047859
wavelet_LHH	glcm	ClusterProminence	− 0.000991
wavelet_HLH	ngtdm	Coarseness	− 0.040312
wavelet_HLL	glcm	Idmn	+ 0.025817
original	shape	Maximum2DDiameterColumn	+ 0.118185
log_sigma_3_0_mm	glcm	Correlation	+ 0.011076
original	glcm	JointAverage	+ 0.095875
wavelet_LHL	firstorder	Skewness	0.047400
wavelet_HHH_	gldm	LowGrayLevelEmphasis	− 0.005149
log_sigma_3_0_mm_3D	firstorder	Mean	− 0.017261
wavelet_LLL	gldm	DependenceNonUniformity	− 0.116212
wavelet_LLL	glcm	Correlation	+ 0.032158

### *Assessing the Utility of radiomics analysis derived from T1-weighted* + *T2-weighted cervical MRI for discriminating between SCC and SCI*

**Figure 5:**
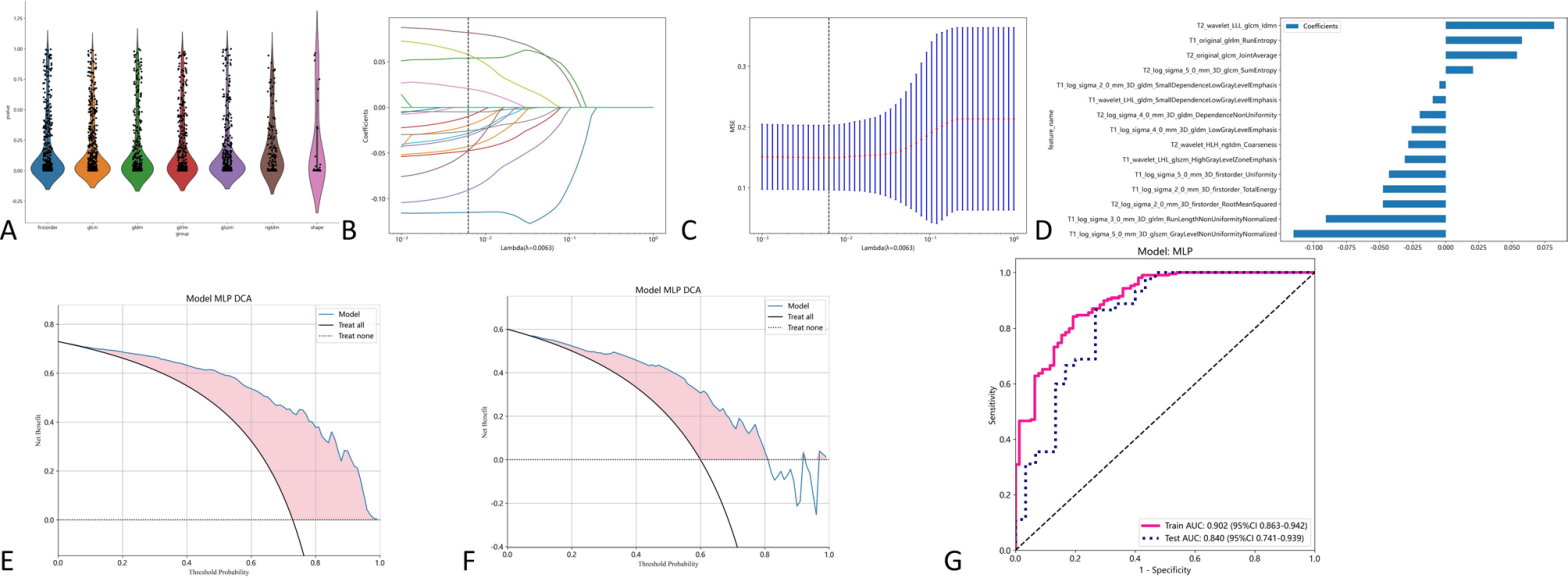
The effectiveness of the T1 + T2 modal. (**A**) Extracted radiomics features. (**B**, **C**) using radiometric methods such as LASSO regression for feature screening. (**D**) Selected radiomics features and feature weights. (**E**) Use MLP machine learning models and filtered features to distinguish the DCA curves of SCC and SCI in the training group. (**F**) Use MLP machine learning models and filtered features to differentiate the DCA curves of SCC and SCI in the test group. (**G**) Use MLP machine learning models and filtered features to distinguish the AUC curves of SCC and SCI.

The pre-fusion method was employed to integrate radiomics features extracted from both the T1-weighted and T2-weighted phases. By merging the 1197 features from the T1-weighted phase with the 1197 features from the T2-weighted phase, a comprehensive set of 2394 features was obtained for subsequent refinement and selection. After screening, 9 radiomics features based on sagittal T1 weighted images and 6 radiomics features based on sagittal T2 weighted images were extracted. The accuracy of using these features to distinguish spinal cord injury and spinal cord concussion in train group is 0.830, AUC is 0.902. The accuracy of using these features to distinguish spinal cord injury and spinal cord concussion in testing group is 0.800, AUC is 0.840. The radiomics feature weights selected based on the T1 + T2 modality of cervical magnetic resonance imaging are presented in Table 4. The effectiveness of using radiomics features based on T2 weighted phase to distinguish SCI and SCC is shown in Fig. 5 and Table 2.

**Table 4:** Radiomics features selected for the T1 + T2 radiomics model.

Image type	Feature class	Feature name	LASSO coefficient (β)
T1_log_sigma_5_0_mm_3D	glszm	GrayLevelNonUniformityNormalized	− 0.114963
T2_log_sigma_4_0_mm_3D	gldm	DependenceNonUniformity	− 0.019632
T2_original	glcm	JointAverage	+ 0.053893
T1_wavelet_LHL	gldm	SmallDependenceLowGrayLevelEmphasis	− 0.009859
T1_log_sigma_3_0_mm_3D	glrlm	RunLengthNonUniformityNormalized	− 0.090545
T2_wavelet_LLL	glcm	Idmn	+ 0.081851
T2_log_sigma_5_0_mm	glcm	SumEntropy	+ 0.020590
T1_log_sigma_2_0_mm	gldm	SmallDependenceLowGrayLevelEmphasis	− 0.004827
T1_original	glrlm	RunEntropy	+ 0.057603
T2_wavelet_HLH	ngtdm	Coarseness	− 0.028292
T1_log_sigma_5_0_mm_3D	firstorder	Uniformity	− 0.042849
T2_log_sigma_2_0_mm_3D	firstorder	RootMeanSquared	− 0.047403
T1_wavelet_LHL	glszm	HighGrayLevelZoneEmphasis	− 0.030920
T1_log_sigma_2_0_mm_3D	firstorder	TotalEnergy	− 0.047352
T1_log_sigma_4_0_mm_3D	gldm	LowGrayLevelRunEmphasis	− 0.025769

### Contrast analysis of effectiveness

**Figure 6:**
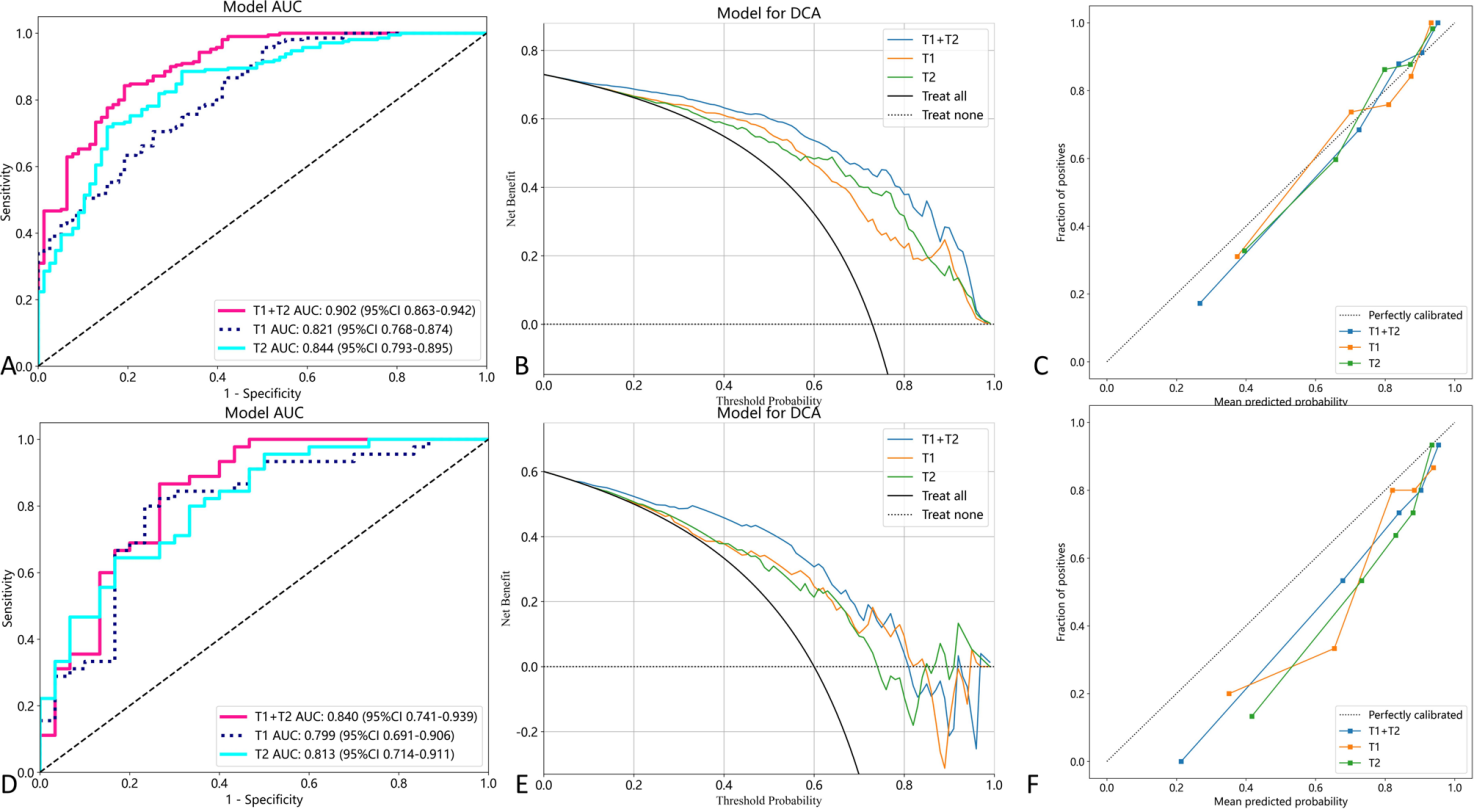
AUC comparison. (**A**) The AUC curves of three modalities in the training group. (**B**) The DCA curves of three modalities in the training group. (**C**) The DCA calibration curves of three modalities in the training group. (**D**) The AUC curves of three modes in the test group. (**E**) The DCA curves of three modes in the test group. (**F**) The DCA calibration curves of three modes in the test group.

The DeLong test was utilized to compare the Area Under the Curve (AUC) of the T1, T2, and combined T1 + T2 modalities for their ability to differentiate between Spinal Cord Injury (SCI) and Squamous Cell Carcinoma (SCC). Comparative analysis revealed that, within the training group, the combined T1 + T2 modality significantly outperformed the individual T1 and T2 modalities, with no significant difference observed between the T1 and T2 modalities alone. However, in the testing group, there was no statistically significant difference in the AUCs across the three modalities. The DeLong test results are presented in Table 5 and Fig. 6.

**Table 5:** AUC comparison.

T1 vs T2	T1 + T2 vs T1	T1 + T2 vs T2	Task
0.46	< 0.01	< 0.01	Train
0.82	0.24	0.54	Test

## Discussion

In our research, the conclusive multimodal model, encompassing T1 and T2 modalities, exhibited an accuracy rate of 0.800 and an AUC value of 0.840 in discriminating between spinal cord injury and spinal cord concussion within the test group. This finding indicates that multimodal radiomics technology, particularly when applied to cervical magnetic resonance imaging, holds promise in enabling the early identification of spinal cord injury and spinal cord concussion among patients. This finding also lays a research foundation for the application of radiomics techniques in the domain of spinal cord injury diagnostics.

The recovery patterns following diverse cervical spinal cord injuries, encompassing concussions, exhibit a wide range of complexities and are often unpredictable in their course^22^. Several complicating elements, including concurrent brain trauma, discomfort, the need for tracheal intubation, and the use of sedatives, can hinder precise evaluations of SCC and SCI, thus making prediction more difficult^23^. Prompt identification of individuals exhibiting symptoms of cervical spinal cord injury and differentiation of those whose condition may not necessitate specialized intervention for spinal cord concussion are critical for informed clinical decision-making^24^.

Currently, the reliance on cervical magnetic resonance imaging (MRI) to discern whether patients with spinal cord injury symptoms have experienced a concussion or other forms of spinal trauma chiefly depends upon subjective and qualitative interpretation of MRI findings, encompassing local signal abnormalities and signs indicative of bleeding and edema^25^. Cervical MRI represents a conventional diagnostic modality for evaluating spinal cord injuries and has been extensively explored for its capacity to gauge injury severity and anticipate outcomes^26^. Nevertheless, traditional cervical MRI scanning encounters certain limitations, particularly in delineating the extent and scope of lesions and in prognostic evaluation. Age-related and occupational factors contribute to degenerative alterations in the human cervical spine, manifesting as marginal osteophyte formation, disc herniation, ossification, and hypertrophy of the ligamentum flavum, along with segmental posterior longitudinal ligament changes—all potentially leading to spinal canal stenosis and reduced buffering space^27^. When external forces are applied to the cervical spine, these pre-existing conditions can exacerbate the degree of the lesion through inward folding of thickened ligaments and protruding intervertebral discs. Furthermore, compression of the spinal cord under these circumstances can readily precipitate acute cervical spinal cord injury, even in the absence of fracture or dislocation, with such injuries potentially eluding positive detection on cervical MRI during early stages^28^.

Therefore, in addition to developing enhanced quantitative imaging protocols, identifying cervical MRI biomarkers capable of differentiating between spinal cord concussions and other urgently treatable spinal cord injuries holds significant clinical relevance^29^. However, literature on the application of MRI biomarkers for predicting injury outcomes remains scarce. Scholars have harnessed deep learning techniques to analyze cervical MRI data; one study reported using such methods based on cervical MRI to differentiate between spinal cord injury and concussion with a commendable accuracy of 0.71^12^. Another research group utilized EfficientNetB2 to ascertain the presence of cervical spinal cord compression due to stenosis on cervical MRI, achieving an accuracy and AUC of 0.81^30^. The author initially crafted a biomarker premised on cervical MRI radiomics to discern between spinal cord injury and concussion, which was employed to anticipate the short-term recovery potential of patients with cervical spinal cord injury symptoms, yielding gratifying results. The biomarkers extracted through radiomics analysis based on cervical MRI offer valuable assistance in clinical decision-making processes.

In our study, the radiomics features log_stigma_5_0_mmD_glszmGrayLevelNonUniformity Normalized in the T1 phase and log_stigma_4_0_mm3D_gldmDependenceNonUniformity in the T2 phase exhibit the highest weights. The former measures the uniformity of gray level distribution in the image, while the latter assesses the uniformity of dependency patterns. This suggests that grayscale and frequency information from magnetic resonance imaging may potentially indicate the presence of spinal cord oscillation or spinal cord injury. However, further research is required to validate these findings.

This study does have some limitations. Firstly, despite being a multi-center effort, the sample size is relatively small, which may impact the generalizability of the findings. Secondly, the region of interest (ROI) analysis in this study combines manual and semi-automatic techniques; a fully automated approach could be explored in future research for improved accuracy and efficiency. Furthermore, the study’s patient sample was predominantly drawn from the Asian population. This concentration may introduce certain biases into the outcomes.

## Conclusion

Our research indicates that multimodal radiomics techniques utilizing cervical magnetic resonance imaging can effectively diagnose the presence of cervical spinal cord injury or spinal cord concussion.

## Data Availability

The datasets generated and/or analysed in this study are available from the corresponding author on reasonable request.

## Abbreviations

SCIWORA: Spinal cord injury without radiographic abnormality
MRI: Magnetic resonance imaging
SCI: Spinal cord injury
SCC: Spinal cord concussion
ROI: Regions of interest
ICC: Intraclass correlation coefficient
AUC: Area under the curve
